# Hybridization between an endangered freshwater fish and an introduced congeneric species and consequent genetic introgression

**DOI:** 10.1371/journal.pone.0212452

**Published:** 2019-02-14

**Authors:** Hiroki Hata, Yohsuke Uemura, Kaito Ouchi, Hideki Matsuba

**Affiliations:** 1 Graduate School of Science and Engineering, Ehime University, Matsuyama, Ehime, Japan; 2 Department of Biology, Faculty of Science, Ehime University, Matsuyama, Ehime, Japan; Uppsala Universitet, SWEDEN

## Abstract

Artificial transplantation of organisms and consequent invasive hybridization can lead to the extinction of native species. In Matsuyama, Japan, a native bitterling fish, *Tanakia lanceolata*, is known to form hybrids with another bitterling species, *T*. *limbata*, which was recently introduced from western Kyushu, Japan. These bitterlings spawn in the gills of two freshwater unionid species, *Pronodularia japanensis* and *Nodularia douglasiae nipponensis*, which have rapidly declined on the Matsuyama Plain in the past 30 years. To gauge the effect of invasive hybridization, we determined the genetic introgression between *T*. *lanceolata* and *T*. *limbata* and analyzed the morphology of these species and their hybrids to infer their niche overlap. We collected adult individuals of *Tanakia* spp. and genotyped them based on six microsatellite loci and mitochondrial cytochrome *b* sequences. We analyzed their meristic characters and body shapes by geometric morphometrics. We found that 10.9% of all individuals collected were hybrids. Whereas *T*. *lanceolata* were more densely distributed downstream and *T*. *limbata* were distributed upstream, their hybrids were widely distributed, covering the entire range of native *T*. *lanceolata*. The body height and anal fin length of *T*. *limbata* were greater than those of *T*. *lanceolata*, but their hybrids were highly morphologically variable, covering both parental morphs, and were widely distributed in the habitats of both parental species. Hybridization has occurred in both directions, but introduced *T*. *limbata* females and native *T*. *lanceolata* males are more likely to have crossed. This study shows that invasive hybridization with the introduced *T*. *limbata* is a potential threat to the native population of *T*. *lanceolata* via genetic introgression and replacement of its niche in streams.

## Introduction

The introduction of non-native organisms into a habitat can bring about the extinction of related native species through competition and hybridization [1,2]. In rivers in western North America, for example, native cutthroat trout breed with introduced rainbow trout and face local extinctions through competition and displacement by the resulting hybrids, although the two parent species seldom mix with each other in naturally coexisting ranges [3,4]. In Japanese freshwater systems, invasive hybrids of alien and native cyprinid species are causing the decline of the native species [5,6].

Bitterling fishes of the family Cyprinidae inhabit rivers, agricultural ditches, and small ponds, and breed by depositing eggs in the gills of freshwater bivalves [7,8]. In Japan, 15 of the 16 native bitterling species and subspecies are listed on the Japanese Red List, facing extinction crises due to multiple stresses, the most critical of which are habitat loss from urbanization, river improvement, and the consequent decline of unionid bivalve populations [9,10,11,12]. The introduction of alien species is another factor negatively impacting native species. The rosy bitterling *Rhodeus ocellatus ocellatus* was introduced to Japanese waters from China in 1942 [13]; consequent competition for habitat and breeding sites (unionid species) along with invasive hybridization caused the extinction of most populations of native *R*. *ocellatus kurumeus* [14]. In western Japan, *Tanakia limbata* has been artificially transplanted to some rivers outside its native range, where it generates invasive hybrids with the native congeneric species *T*. *lanceolata* [15]. *Tanakia limbata* was introduced to Ehime Prefecture in the 1970s [16] and now competes with two other bitterlings there, the native *T*. *lanceolata* and the exotic *R*. *ocellatus ocellatus*, for the use of the same unionid species as breeding substrata [15]. Populations of the unionid mussels, *Pronodularia japanensis* and *Nodularia douglasiae nipponensis*, which serve as spawning sites for bitterlings on the Matsuyama Plain, have decreased rapidly over the past 30 years because of habitat degradation [17]. Consequently, *T*. *lanceolata* populations have also decreased in the past two decades [18]. The decline of suitable breeding substrata may also force bitterlings to engage in group spawning by multiple males and females instead of pair spawning, a behavior that has been observed in the bitterling *Rhodeus ocellatus* [19]. In group spawning, different bitterling species may spawn simultaneously and hybridize incidentally with each other [1].

*Tanakia lanceolata* and *T*. *limbata* diverged about 10 million years ago [20]. They produce fertile hybrids [21], but the survival rate of these hybrids in the post-larval stage is lower than that of purebred fish, suggesting some degree of postzygotic isolation [22]. Prezygotic isolation has also likely been established through sexual isolation between hybridizing fishes because of a decline in parental fitness of that breed with other species [23,24]. In fact, *T*. *lanceolata* and *T*. *limbata* are somewhat segregated by habitat and prefer different breeding substrates within a river: *T*. *lanceolata* prefers faster-flowing waters and spawns mainly on unionids in the thalweg section, whereas *T*. *limbata* lives and spawns near the banks of rivers, where the flow is weaker [25]. Male *T*. *limbata* will defend a unionid breeding territory against *T*. *lanceolata* as well as other *T*. *limbata* males [26,27].

The fitness of hybrid individuals determines the consequences of hybridization, (i.e. whether it enhances speciation by adaptive radiation through transgressive segregation [28,29], causes the extinction of either parent, or leads to the fusion of the parental species [30,31]). In theory, the fitness of hybrids is strongly affected by the phylogenetic distance between its two parent species and the variety of ecological niches that are available [32], but it is necessary to accumulate more empirical studies about the phylogenetic distances between hybridizing species, the traits and niche preference of hybrid individuals, and the consequences of hybridization to construct a general rule about hybrid fitness.

To this end, we classified individuals as *T*. *lanceolata*, *T*. *limbata*, or their hybrid using microsatellite nuclear DNA markers and mitochondrial cytochrome *b* sequences. We also analyzed the direction of hybridization using both nuclear and mitochondrial genes. Finally, we examined the body shapes, meristic characters, and longitudinal distribution within streams of *T*. *lanceolata*, *T*. *limbata*, and their hybrids.

## Materials and methods

### Ethics statement

The field sampling and sample treatment were conducted in accordance with the "Guidelines for the use of fishes in research" by the Ichthyological Society of Japan (http://www.fish-isj.jp/english/guidelines.html). All animal experiments were approved by the Ethics Committee for Animal Experiments of Ehime University. The experimental procedures were conducted in accordance with the approved guidelines.

### Sampling

**Fig 1:**
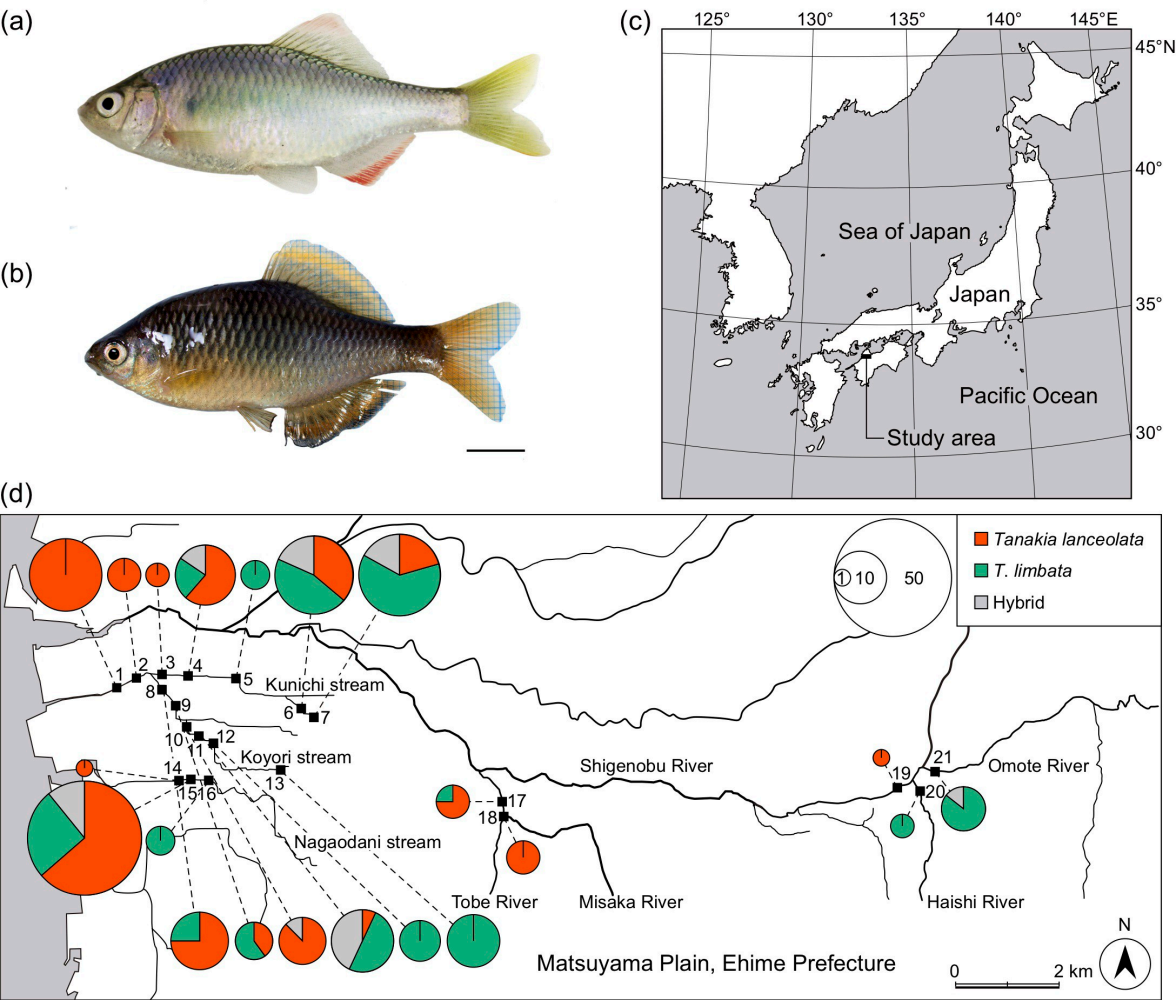
Study species and maps of the study sites on the Matsuyama Plain, Ehime prefecture, Japan, and distribution of native *Tanakia lanceolata*, introduced *T*. *limbata*, and their hybrids. (a) *Tanakia lanceolata* and (b) *T*. *limbata* with the scale bar of 10 mm. (c) Location of the study areas in Japan. (d) Distribution of *T*. *lanceolata* (red), *T*. *limbata* (green), and hybrids of these two species (gray) classified based on six microsatellite markers. Circle size indicates the number of individuals. We have drawn these maps ourselves based on maps provided under CC BY 4.0 by the Geospatial Information Authority of Japan (https://maps.gsi.go.jp).

We collected 211 *Tanakia* individuals at five sites in the Shigenobu River system, 13 sites in the Kunichi River system, and three sites in the Nagaodani stream (33°46' N–33°47' N, 132°42' E–132°53' E) between May 2011 and October 2013 using an electrofishing unit (LR-24, Smith-Root Inc., Vancouver, WA, USA; Fig 1). These sampling sites cover the distribution range of *T*. *lanceolata* and *T*. *limbata* on the Matsuyama plain [15,18]. We collected *Tanakia* individuals from a 50 m section along the river/streams at each site to limit negative effects on the endangered *T*. *lanceolata* population. Ehime Prefecture granted permission to conduct this sampling. The collected specimens were sacrificed by immersion in an ice-water slurry, and their right pelvic fins were removed and fixed in 99.9% ethanol for molecular analysis. We then spread the specimens’ fins with needles and photographed their left sides for morphometric analysis using a digital camera E-520 (Olympus, Tokyo, Japan). We fixed specimens in 10% formalin for 2 weeks, after which they were washed and moved to 70% ethanol for preservation. All of the specimens are stored as voucher specimens at the Department of Biology, Graduate School of Science and Engineering, Ehime University.

### DNA sequencing and genotyping of cytochrome *b*

Clippings (ca. 2 × 2 mm) were taken from the ethanol-preserved fins, and genomic DNA was extracted using the Wizard Genomic DNA Purification Kit following the manufacturer’s protocol (Promega, Madison, WI, USA). We amplified the cytochrome *b* (cyt *b*) region of mtDNA by PCR with the forward primer NEW-FOR, 5’-AGCCTACGAAAAACACACCC- 3’ [33], and the reverse primer cytb-Rev, 5’-GATCTTCGGATTACAAGACC-3’ [34]. The reaction mixture contained 6.05 μL of sterile distilled water, 1.0 μL of 10× ImmoBuffer (Bioline, London, UK), 1.0 μL of dNTP mix (10 mM), 0.3 μL of MgCl_2_ (50 mM), 0.3 μL of each primer (10 μM), 0.05 μL of BIOTAQ HS DNA polymerase (5U/μL, Bioline), and 1.0 μL of DNA template. The reaction protocol consisted of an initial denaturation at 95°C for 10 min; this was followed by 30 cycles of 95°C for 60 s, 58°C for 60 s, and 72°C for 90 s and a final extension at 72°C for 7 min. We obtained sequences for 48 of the 211 collected individuals as follows: PCR products were purified using polyethylene glycol following the protocol of [35] and subjected to direct cycle sequencing with BigDye Terminator version 3.1 (Thermo Fisher Scientific, MA, USA) using the PCR primers. Sequencing followed the ABI recommended protocol. Labeled fragments were sequenced using an ABI 3130 Genetic Analyzer (Thermo Fisher Scientific). Sequences were stored under the accession numbers AB907119–31, AB907133–5, AB907145–8, and AB920289–91. Following sequencing, we defined the mitochondrial genotype of each individual as *T*. *lanceolata* or *T*. *limbata* [15,18].

The remaining 163 individuals were genotyped by PCR-restriction fragment length polymorphism (PCR-RFLP) using the restriction enzyme *Hha*I (TaKaRa Bio, Shiga, Japan), which cuts the cyt *b* fragments of *T*. *lanceolata* and *T*. *limbata* in different locations [15]. *Hha*I produced fragments of 91, 218, 252 and 369 bps for *T*. *lanceolata*, and fragments of 18, 109, 252 and 821 bps for *T*. *limbata* (S1 Fig). We incubated 5.0 μL of the PCR products with 0.2 μL of *Hha*I (10 U/μL), 3.8 μL of sterile distilled water, and 1.0 μL of 10× M buffer overnight. We conducted gel electrophoresis with the reaction products for 40 min at 100 V on 3% agarose gel (NIPPON Genetic, Tokyo, Japan) and genotyped individual fish according to the resulting fragment patterns.

### Genotyping using microsatellite markers

We used six microsatellite loci as markers following our preliminary study [15]: *RC363* and *RC317A*, which were reported from *Rhodeus ocellatus* ssp. [36], and *Rser02*, *Rser03*, *Rser07*, and *Rser10*, which were reported from *R*. *sericeus* [37]. The target sequences were amplified by PCR following the method of [38], and each microsatellite primer was labeled with one of four fluorescent dyes: *RC363* and *Rser07* were labeled with PET, *RC317A* and *Rser03* with FAM, *Rser02* with NED, and *Rser10* with VIC. The reaction mixture contained 3.15 μL of sterile distilled water, 0.5 μL of 10× ImmoBuffer (Bioline), 0.5 μL of dNTP mix (10 mM), 0.15 μL of MgCl_2_ (50 mM), 0.02 μL of forward primer (10 μM), 0.08 μL of reverse primer (10 μM), 0.08 μL of fluorescent dye label (10 μM), 0.025 μL of BIOTAQ HS DNA polymerase (5U/μL, Bioline), and 0.5 μL of DNA template. The reaction consisted of an initial denaturation at 95°C for 10 min; followed by 30 cycles of 95°C for 30 s, 60°C for 45 s, and 72°C for 30 s; 10 cycles of 95°C for 30 s, 53°C for 45 s, and 72°C for 45 s; and a final extension at 72°C for 10 min. We determined the lengths of fragments using an ABI 310 Genetic Analyzer (Thermo Fisher Scientific) and Peak Scanner software version 2.0 (Thermo Fisher Scientific). The number of alleles, expected heterozygosity (*H*_e_), and observed heterozygosity (*H*_o_) were calculated using Genepop on the Web [39,40]. Deviations from Hardy–Weinberg equilibrium (HWE) were also tested using Genepop on the Web [39,40]. Null alleles and their frequencies were determined using Micro-Checker version 2.2.3 [41]. The population genetic structure was analyzed using Structure version 2.3.4 [42] with the following parameters: 100,000 burn-in; 1,000,000 Markov chain Monte Carlo steps; admixture model with independent allele frequencies; and five replicates of each simulation from *K* = 1 to 10 genetic clusters. The optimum *K* value calculated by Structure Harvester version 0.6.94 [43] was 2 (S2 Fig). As the resultant 2 populations corresponded well with the mitochondrial genotypes and morphological characters, we defined the population with a cyt *b* sequence and morphology of *T*. *lanceolata* as purebred *T*. *lanceolata*, and the other population, which possessed the *T*. *limbata* morphology and cyt *b* sequence, was defined as purebred *T*. *limbata*. We defined hybrids as those with more than 20% of their genetic material matching both purebred type [44].

We used NewHybrids [45] to assign each individual to one of six genotypic classes based on the posterior probabilities: the two parental species (P0, P1), first-generation hybrid (F1), second-generation hybrid (F2), backcross of F1 with P0, and backcross of F1 with P1. We used 0.5 as a threshold value for the posterior probability (*q*) with their assignment following [46]. Parameters of NewHybrids were set as follows: without individual or allele frequency prior information; Jeffreys-like prior for both mixing proportions and allele frequencies; 25,000 sweeps of burn-in; and 100,000 iterations of the Markov chain Monte Carlo. To assess the efficiency and accuracy of genotype assignment by NewHybrids, we conducted a simulation method using HYBRIDLAB version 1.0 [44,47]. A total of 100 simulated hybrids of each hybrid class were generated based on the allelic frequencies of 81 *T*. *lanceolata* individuals and 68 *T*. *limbata* that were identified as purebreds in the previous Structure analysis, with a cut-off value of 0.9 for the ancestry coefficient. This dataset was analyzed by NewHybrids to assign each individual to a genotypic class with *q* = 0.5 to estimate the accuracy and efficiency of the assignment.

### Distribution characteristics

To clarify the longitudinal distribution of bitterlings in the Kunichi, Koyori, and Nagaodani streams, we performed a generalized linear mixed model (GLMM) analysis with the number of individuals of each species/hybrid and the number of other individuals as response variables, distance from the river mouth as a fixed factor, and the stream as a random factor. GLMM analysis was conducted using the glmmML 1.0.3 package in R 3.5.0 [48].

### Analysis of external morphology

**Fig 2:**
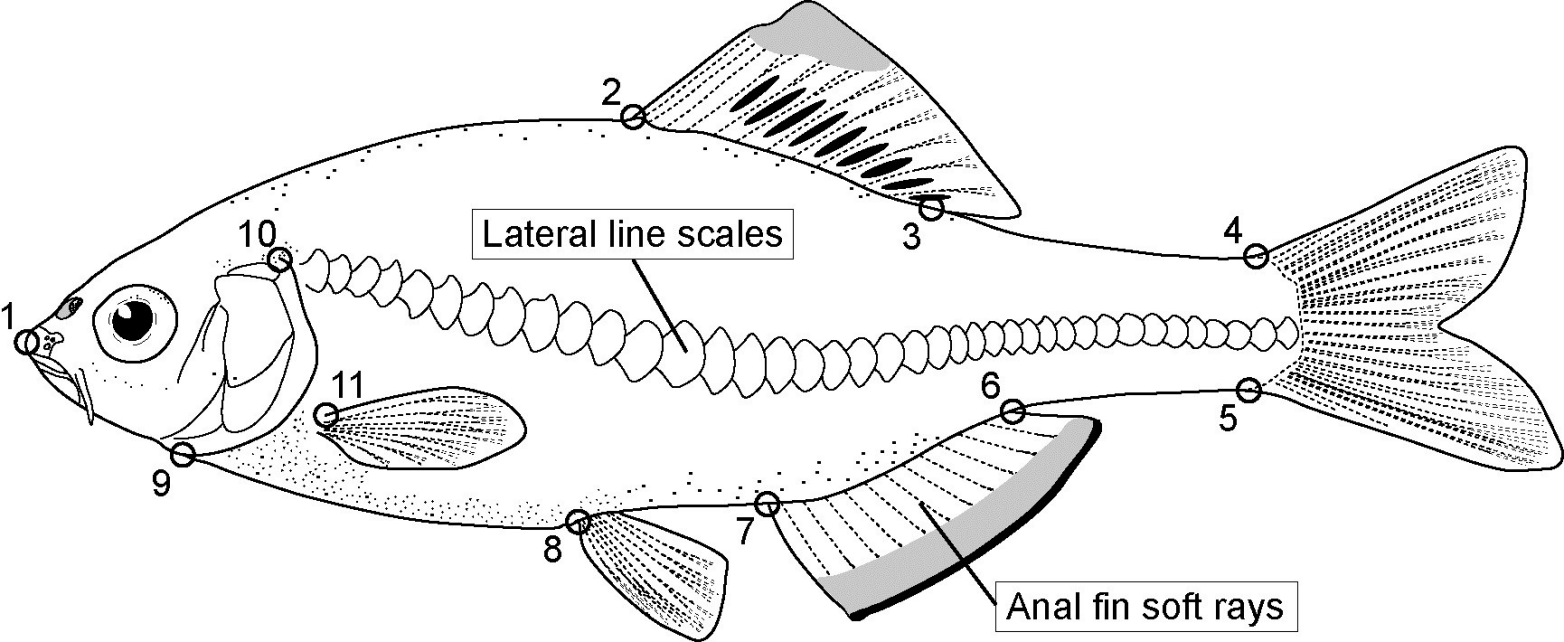
Body shape landmarks for geometric morphometrics. (1) Anterior tip of snout, (2 and 3) anterior and posterior insertion of the dorsal fin, (4 and 5) upper and lower insertion of the caudal fin, (6 and 7) posterior and anterior insertion of the anal fin, (8) insertion of the pelvic fin, (9 and 10) ventral and dorsal insertion of the operculum on the profile, and (11) dorsal insertion of the pectoral fin. Number of lateral line scales and number of anal fin soft rays are observed as meristic characters.

We measured the meristic characters of our bitterling specimens and conducted geometric morphometrics of body shapes to distinguish *T*. *lanceolata*, *T*. *limbata*, and their hybrids. The number of lateral line scales (LLS) and the number of anal fin soft rays (AFR) were used as meristic characters. *Tanakia lanceolata* has 36 to 39 LLS, whereas *T*. *limbata* has 32 to 36; *T*. *lanceolata* has 8 to 10 AFR, and *T*. *limbata* has 9 to 12 [49]. To compare body-shape morphometrics, we placed 11 landmarks on specimen photos in tpsDig2 version 2.32 [50](Fig 2). We conducted a Procrustes analysis based on the coordinate data of the landmarks; this was followed by a principal component analysis (PCA) on the resultant data with MorphoJ [51]. We compared the PC1 and PC2 of bitterling body shapes with a permutational MANOVA (PERMANOVA) using the function Adonis in the package vegan 2.5–2 in R version 3.5.0. We conducted pairwise comparisons using the pairwise.perm.manova function of the RVAideMemoire package version 0.9-69-3.

## Results

### Genetic analyses

**Fig 3:**
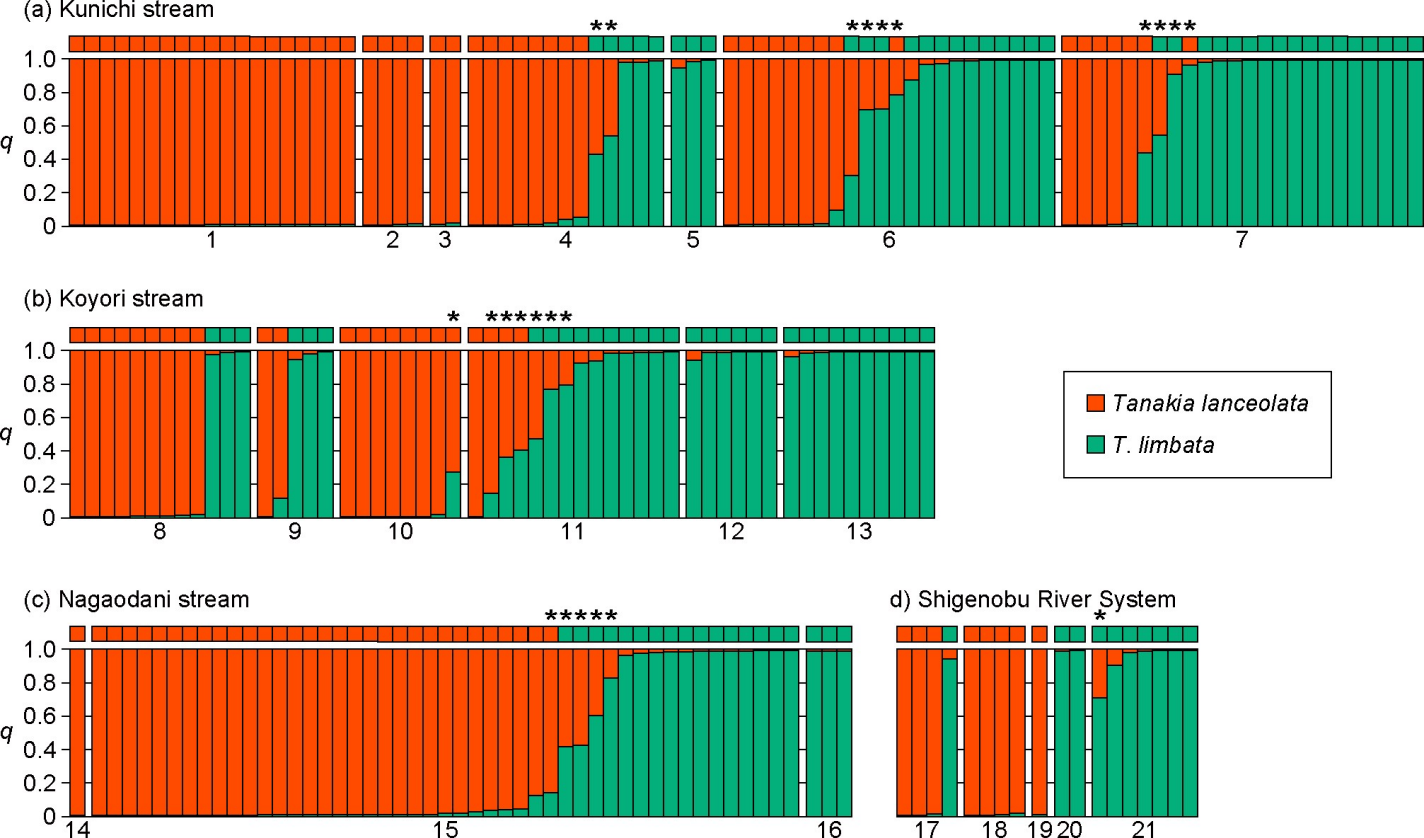
Results of population genetic analysis of *Tanakia lanceolata*, *T*. *limbata*, and their hybrids in streams in Ehime, Japan, based on six microsatellite loci. Squares above bars indicate mitochondrial cytochrome *b* genotypes. Asterisks above squares indicate hybrid individuals. Numbers indicate the locality as shown in Fig 1.

We determined the genotypes of 211 adult *Tanakia* individuals using six microsatellite loci. The results given by Structure and NewHybrids were largely consistent (Fig 3 and S1 and S2 Tables). Therefore, we used the NewHybrids results in the following analyses. Of the 211 subjects, 104 were classified as *T*. *lanceolata*, and all of these had *T*. *lanceolata* mitochondrial cyt *b* sequences; 85 were classified as *T*. *limbata*, and only one of these had the *T*. *lanceolata* cyt *b* type, most likely pointing to that individual being descended from a hybrid. The remaining 22 subjects were hybrids of the two species. The *T*. *lanceolata* cyt *b* sequence was found in 7 of these, whereas the other 15 had the *T*. *limbata* cyt *b* sequence. These hybrid individuals were further classified as 19 F2 individuals and 3 ambiguous hybrids. However, efficiency to distinguish genetic classes among hybrids were limited (S3 Table), and thus we do not distinguish them and instead use a category, hybrid.

### Distribution in streams

**Fig 4:**
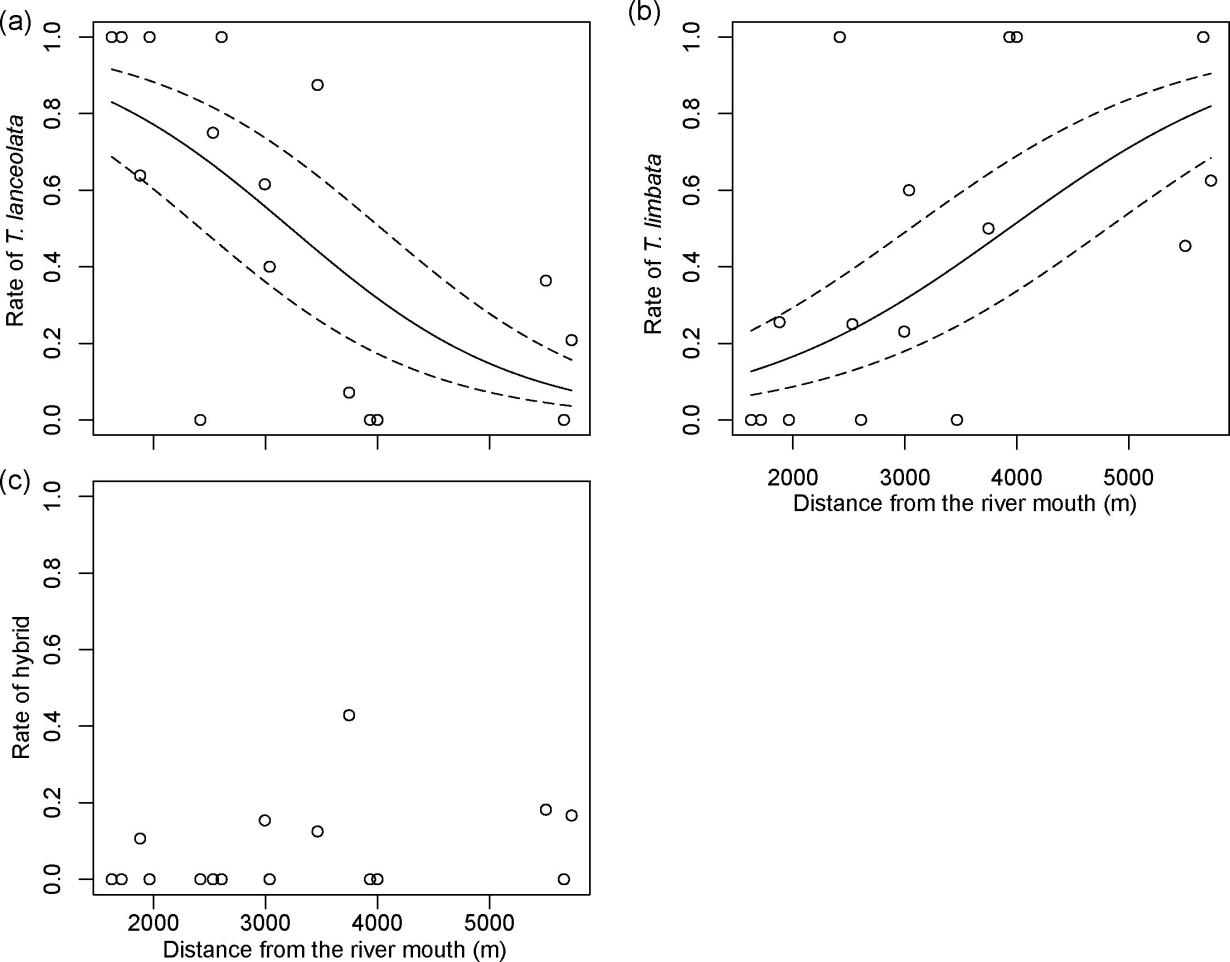
Longitudinal distribution of *Tanakia lanceolata*, *T*. *limbata*, and their hybrids in study streams. (a) *Tanakia lanceolata*, (b) *T*. *limbata*, and (c) their hybrids. Solid curves indicate estimated values based on the result of a GLM without any variance among streams, and dotted curves indicate estimated values based on a GLMM with variance among streams.

Hybrids of *T*. *lanceolata* and *T*. *limbata* were found in the Kunichi, Koyori, and Nagaodani streams and at one site in the Omote River. We found that *T*. *lanceolata* were more densely distributed in the lower reaches of the Kunichi and Nagaodani streams (GLMM, *P* < 0.001; Fig 4A), whereas *T*. *limbata* were concentrated further upstream (GLMM, *P* < 0.001; Fig 4B). Hybrids occurred throughout the stream systems (GLMM, *P* > 0.05; Fig 4C).

### Morphological characteristics

**Fig 5:**
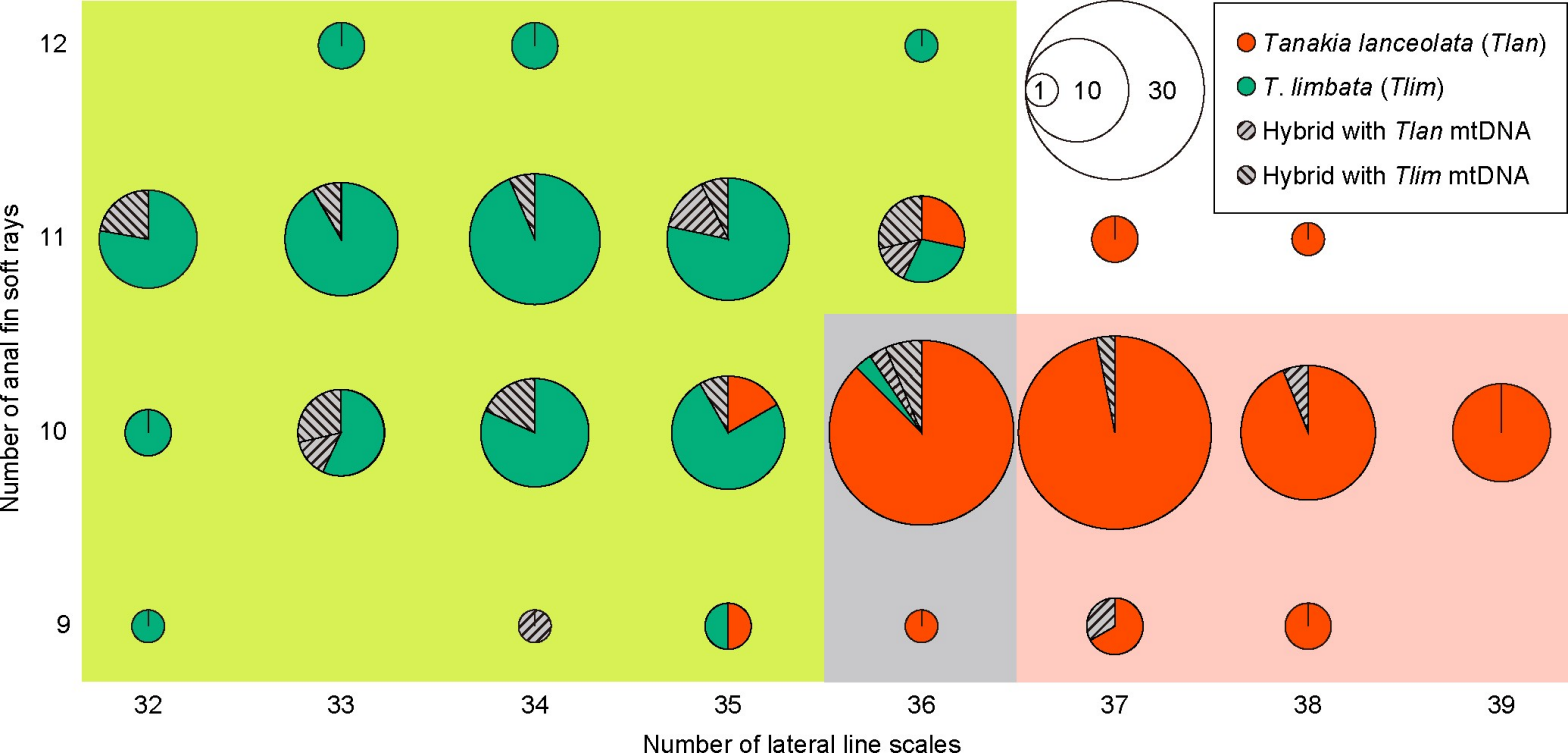
Meristic characters and genotypes of *Tanakia lanceolata*, *T*. *limbata*, and their hybrids. Circle size indicates the number of individuals.

We found meristic characters consistent with *T*. *lanceolata* (36–39 LLS and 9–10 AFR) in 92 (88.5%) of the 104 individuals genetically determined to be purebred *T*. *lanceolata*, whereas 5 (4.8%) had characters unique to *T*. *limbata* (Fig 5). On the other hand, all individuals genetically identified as purebred *T*. *limbata* had the meristic characters of *T*. *limbata* (32–36 LLS and 9–12 AFR). Of the 23 specimens identified as hybrids, 3 (13.0%) had characters unique to *T*. *lanceolata*, 3 (13.0%) had intermediate characters (36 LLS and 9–10 AFR), and the other 17 (73.9%) had characters unique to *T*. *limbata*.

**Fig 6:**
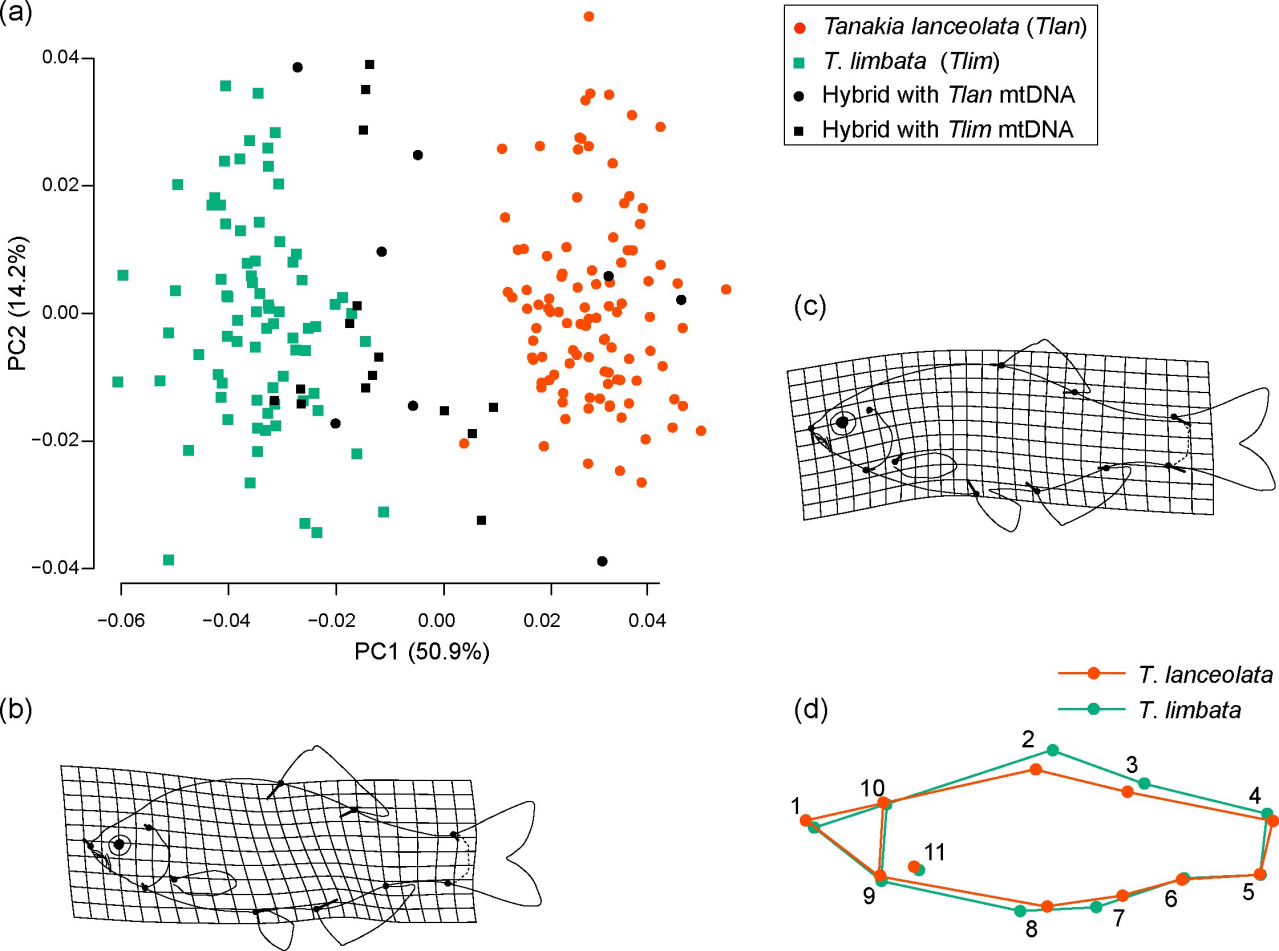
Differences in the body shapes of *Tanakia lanceolata*, *T*. *limbata*, and their hybrids. (a) Variations in body shape based on geometric morphometrics. Genotyping was conducted in NewHybrids based on six microsatellite loci. Circles indicate individuals with *T*. *lanceolata* mitochondrial genotype; squares indicate *T*. *limbata* mitochondrial genotypes. (b) Variations in shape along the PC1 axis. (c) Variations in shape along the PC2 axis. The lines in (b and c) indicate shape changes following the plot shifts in (a) with 0.1 unit in the positive PC direction. (d) Comparison between individuals with the maximum (*T*. *lanceolata* morph) and minimum (*T*. *limbata* morph) PC1 scores.

Geometric morphometric analysis showed that the body shape of *T*. *lanceolata*, *T*. *limbata*, and their hybrids differed significantly (PERMANOVA, *R*^2^ = 0.69, *P* < 0.001). The groups had significantly different PC1 values (pairwise PERMANOVA, *P* < 0.001 for all the pairs): they were positive in *T*. *lanceolata*, negative in *T*. *limbata*, and their hybrids covered the full range of both parental species (Fig 6A). The difference in the body shapes of *T*. *lanceolata* and *T*. *limbata* was largely derived from four landmarks (2, 3, 7, and 11) reflecting body height, anal fin length, and the position of the pelvic fin (Figs 2 and 6B). Of the 23 hybrids, 14 (66.7%) had negative PC1 values, suggesting that these had body shapes to that of *T*. *limbata*. There was no difference in the body shape of hybrid individuals with mitochondrial genotypes of *T*. *lanceolata* and *T*. *limbata* (PERMANOVA, *P* > 0.05). Note that no significant differences were detected between females and males in *T*. *lanceolata*, *T*. *limbata*, or their hybrids.

### Relationship between morphology and distribution in streams

**Fig 7:**
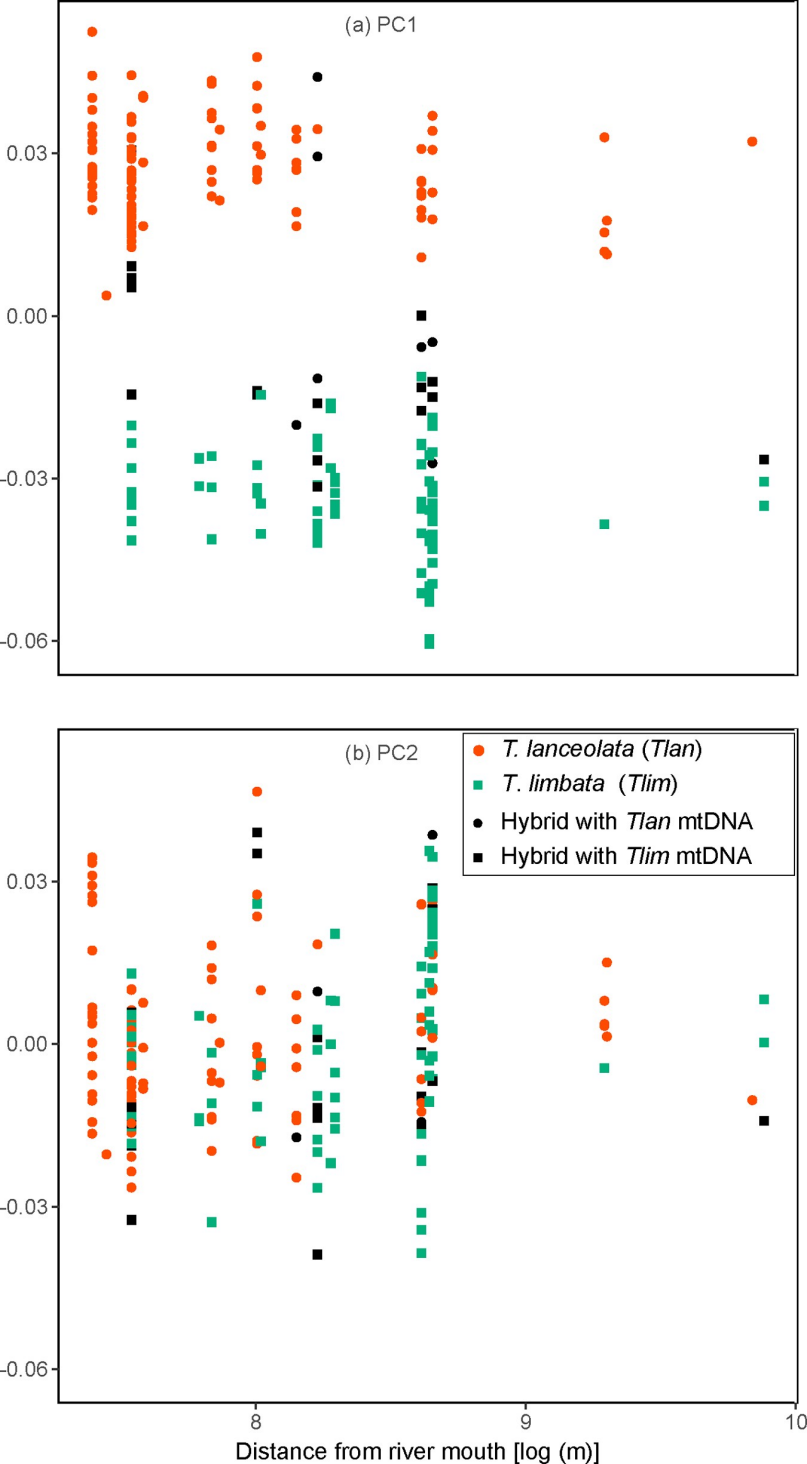
Relationships between body shape and longitudinal distribution in rivers of *T*. *lanceolata*, *T*. *limbata*, and their hybrids. (a) Relationship between distance from the river mouth and PC1 values of geometric morphometrics of body shape; (b) relationship between distance from the river mouth and PC2 values.

No relationship was detected between the body shape (PC1 or PC2 of geometric morphometrics) and collection site (distance from the river mouth; GLM, *P* > 0.05 for *T*. *lanceolata*, *T*. *limbata*, and their hybrids; Fig 7).

## Discussion

### Morphological differences and habitat niches in streams

In terms of the longitudinal distribution of bitterlings in the study streams, *T*. *lanceolata* were found primarily downstream, whereas *T*. *limbata* preferred upstream waters. In the Harai River in central Japan, where these two bitterlings are sympatric, their habitat is segregated within the river: *T*. *limbata* is distributed near the banks, where water is slow flowing and shallower, and *T*. *lanceolata* is widely distributed in the middle of the river [25]. In another sympatric locality in northern Kyushu, *T*. *limbata* are found upstream of *T*. *lanceolata* [52]. We found similar habitat segregation in the three streams in this study, with *T*. *limbata* distributed primarily upstream, where water flow was slower. Morphometric analysis showed that *T*. *limbata* had greater body height than did *T*. *lanceolata*, which is consistent with previous results given by the direct measurement of the body depth of *Tanakia* spp. in the Harai River [25]. Body height correlates negatively with swimming speed in cyprinid fishes [53]. The taller *T*. *limbata* experiences more drag than *T*. *lanceolata* and thus prefers habitats with slower-flowing water. Hybrid individuals with a *T*. *limbata*-like body shape may also prefer slower flow, but hybrids with an intermediate body shape may expand their distribution to habitats with faster water flow. The body shape of hybrids can be intermediate between parental species, as in the hybrids of another pair of cyprinid fishes, *Rutilus rutilus* and *Abramis brama*, in an Irish lake [54], but this is far from the only possibility. Hybrids can have a wide range of body shapes extending to the morphs of both parental species and sometimes exceeding the range of their parent species (through transgressive segregation), as in African cichlids [28]. Hybrids in this study had a wide range of both meristic characters and body shapes, covering the morphs of both parental species, and were distributed both upstream and downstream. Kunichi, Koyori, and Nagaodani streams are inhabited by the unionids *Nodularia douglasiae nipponensis*, *Pronodularia japanensis*, and *Sinanodonta* spp. However, the ranges of distribution of these unionids, as well as their population densities, have rapidly declined in the past 30 years. Only a small *P*. *japanensis* population remains in the middle reaches where the habitats of *T*. *lanceolata* and *T*. *limbata* overlap [17]. Therefore, in the breeding season (from March to July for *T*. *lanceolata* and from March to September for *T*. *limbata*), adult fishes gather in the midstream for reproduction [15,52]. Thus, the habitat segregation observed between *T*. *lanceolata* and *T*. *limbata* seems to be caused by different preferences for habitat based on different body shapes, and annual migration.

### Cause of hybridization

In this system, it is difficult to accurately distinguish genetic classes among hybrids based on six microsatellite markers, but a high rate of F2 hybrids, 19 of 23 hybrid individuals (82.6%), was detected. This result suggests that the genetic introgression proceeds from this invasive hybridization. Although some bitterling fish species prefer different freshwater mussel species [25,55], *T*. *lanceolata* and *T*. *limbata* choose the same species of unionid [25] and sometimes spawn their eggs simultaneously in the same individual mussels. Thus, the rapid decline of the unionid population in their habitats causes overcrowding of bitterling fishes and accelerates competition for breeding substrata. Bitterling males are known to use alternative reproductive tactics depending on the availability of females and the breeding substratum (mussels); they may engage in pair spawning on a mussel territory, sneaking toward a pair, or group spawning with multiple males [19,56,57,58]. When the density of bitterlings is much higher than that of unionids, territoriality collapses, and sneaking and group spawning become dominant [19,59,60]. In both of these strategies, different bitterling species spawn simultaneously and may incidentally hybridize with each other [1,61]. In fact, *T*. *lanceolata* and *T*. *limbata* have been observed breeding simultaneously on the same *Pronodularia japanensis* at our study site (Matsuba, Ouchi, Hata, personal observations). The limited availability of suitable breeding substrata due to the rapid decline of mussel populations and the concentration of the remaining unionid population in the midstream, where the ranges of the two bitterling species overlap, increases the frequency of encounters between native *T*. *lanceolata* and *T*. *limbata*. Hybridization occurred in both directions but was skewed toward female *T*. *limbata–*male *T*. *lanceolata* crosses. This bias is likely due to the rarity of introduced species during the colonizing period; in group spawning, *T*. *lanceolata* eggs might be encountered and fertilized more frequently by sperm of the more common *T*. *lanceolata*.

### Conservation perspective

On the Matsuyama Plain, native *T*. *lanceolata* are at risk due to competition for breeding substrata and continuous genetic introgression with introduced *T*. *limbata*. *Tanakia lanceolata* currently shares its entire range and breeding substratum with *T*. *limbata* and their hybrids, and is in competition with both. A necessary first step in the conservation of *T*. *lanceolata* is the construction of a protected area. The midstream of the stream system can be a focal area [62], where unionids survive and from which *T*. *limbata* and hybrids can be eliminated and their further immigration restricted. In Japan, the endangered bitterling fishes, *Rhodeus ocellatus kurumeus*, *Pseudorhodeus tanago*, and *Acheilognathus longipinnis*, are conserved in irrigation reservoirs scattered around the native range, backwater pools of a river, and irrigation creeks, respectively [63,64]. At the same time, conservation of declining mussels throughout the stream systems is crucial as a part of global efforts to conserve freshwater mussels [65].

## Supporting information

S1 Table Genotyping of *Tanakia* individuals in Matsuyama, Ehime, Japan, based on six microsatellite loci using Structure, NewHybrids, and mitochondrial cytochrome *b* sequences. (XLSX)

S2 Table Allelic variability at six microsatellite loci in populations of *Tanakia lanceolata*, *T*. *limbata*, and their hybrids in Matsuyama. *H*_o_, observed heterozygosity; *H*_e_, expected heterozygosity. HWE, the Hardy–Weinberg equilibrium. * denotes significant deviation from HWE after Bonferroni correction.
(XLSX)

S3 Table Efficiency and accuracy of genotype assignment on our data using by NewHybrids. (XLSX)

S1 Fig An example of gel image used to determine the cyt *b* haplotype of *Tanakia lanceolata* (a above the lanes) and *T*. *limbata* (i above the lanes). (TIF)

S2 Fig Delta *K* values calculated by the Evanno method using structure Harvester version 0.6.94. (TIF)
