# Supplementary figures and images for: Hybridization between an endangered freshwater fish and an introduced congeneric species and consequent genetic introgression
Source: PLoS One. 2019 Feb 14;14(2):e0212452. doi: 10.1371/journal.pone.0212452 (PMC6375628; doi:10.1371/journal.pone.0212452)

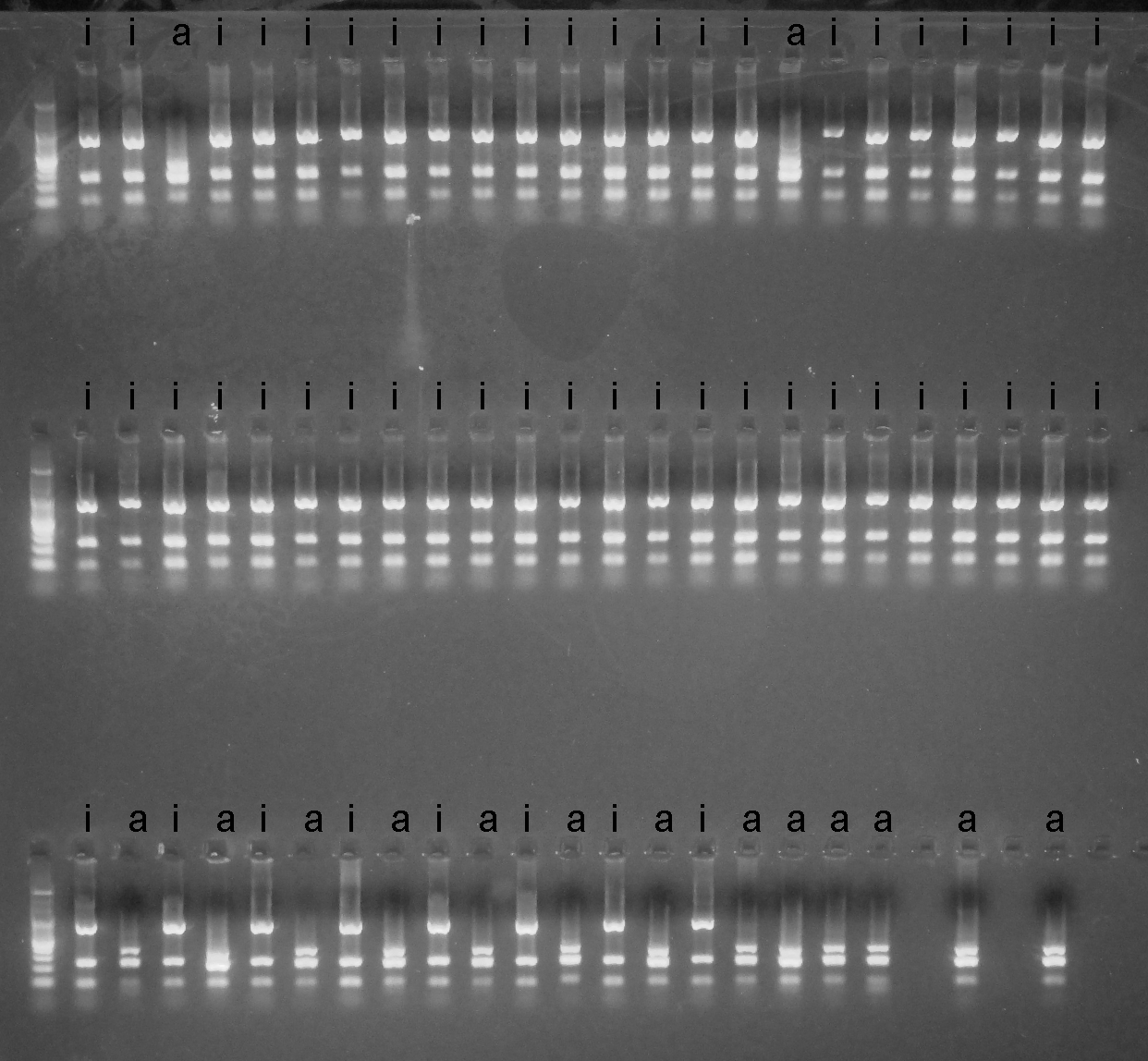

Supplement: S1 Fig — (TIF) [file pone.0212452.s004.tif]

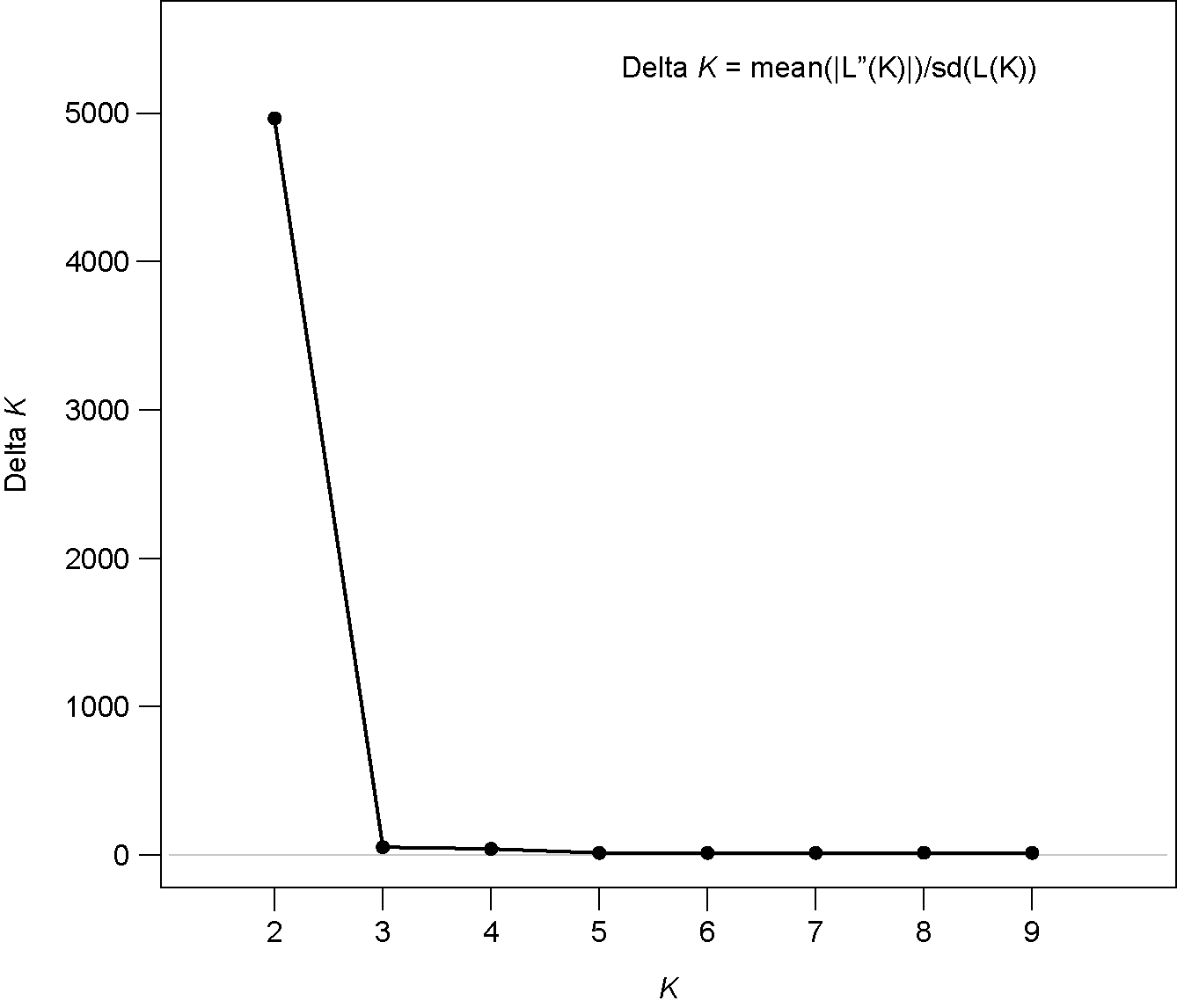

Supplement: S2 Fig — (TIF) [file pone.0212452.s005.tif]
